# Initial Hemorrhagic Pericardial Effusion Evolving Into Perimyocarditis: An Atypical Early Presentation of Eosinophilic Granulomatosis With Polyangiitis

**DOI:** 10.7759/cureus.88155

**Published:** 2025-07-17

**Authors:** Samah A Almatrafi, Salma M Quqandi, Samaher J Ismail, Khalid A Alghamdi

**Affiliations:** 1 Internal Medicine, King Abdulaziz Medical City, Ministry of National Guard-Health Affairs, Jeddah, SAU; 2 Internal Medicine, King Abdullah International Medical Research Center, Jeddah, SAU; 3 Rheumatology, King Abdulaziz Medical City, Ministry of National Guard-Health Affairs, Jeddah, SAU; 4 Rheumatology, King Abdullah International Medical Research Center, Jeddah, SAU

**Keywords:** eosinophilic granulomatosis with polyangiitis (egpa), hemorrhagic pericarditis, myopericarditis, pericardial fluid, perimyocarditis

## Abstract

Eosinophilic granulomatosis with polyangiitis (EGPA) is a form of multisystemic necrotizing vasculitis that has various manifestations. Cardiac involvement is one of the indications of poor prognosis, occurring with different reported prevalence. Due to the heterogeneity of EGPA presentation, its diagnosis relies on clinical condition supported by different investigations and classification criteria. However, large hemorrhagic pericardial effusion is a rare occurrence in EGPA, raising a diagnostic challenge with a wide differential diagnosis. EGPA can also affect other layers of the heart, making its development to perimyocarditis a warning for the progressive nature of the disease. We report a case of EGPA in a young female patient who presented initially with large hemorrhagic pericardial effusion, and later developed perimyocarditis.

## Introduction

Eosinophilic granulomatosis with polyangiitis (EGPA) is a rare autoimmune disease causing necrotizing inflammation of small-to-medium vessels [1]. It is characterized clinically by a triad of eosinophilia, refractory asthma, and systemic vasculitis affecting multiple organs, making its diagnosis challenging [1]. Recognition of EGPA has increased over the last few years, and cardiac manifestations are reported in 16-34% of cases, presenting in various forms [1,2]. Early recognition of such manifestations in EGPA is crucial, as it is the leading cause of mortality, accounting for approximately 50% of disease-related deaths [3]. Pericardial effusion in EGPA is recognized in the literature, typically with serous fluid containing eosinophilic cells [4]. However, large hemorrhagic pericardial effusion is extremely rare, and it may mimic other conditions such as malignancy, tuberculosis (TB), or other vasculitis, posing a diagnostic challenge [5]. The extension of cardiac involvement in EGPA can reach the myocardium, reflecting the progressive and heterogeneous nature of the disease [5]. We report a rare case of EGPA initially presenting with a large hemorrhagic pericardial effusion, later complicated by eosinophilic perimyocarditis. This case emphasize the importance of considering EGPA in young patients with unexplained hemorrhagic pericardial effusions and peripheral eosinophilia.

## Case presentation

A 17-year-old Saudi female patient, recently diagnosed with asthma and allergic rhinitis, had multiple presentations due to chest pain. During her first presentation to the emergency department (ED), she reported a two-week history of non-specific symptoms of fatigue and night sweats, followed by a two-day history of sharp central chest pain that improved when leaning forward. Initial evaluation showed large pericardial effusion, seen in echocardiogram, with bilateral lung consolidations, which necessitated an admission under cardiology as a case of pericardial effusion and presumed community-acquired pneumonia. An emergency pericardiocentesis was done draining bloody exudative fluid. Cytological analysis revealed no abnormal cells, with neutrophils present and no eosinophils identified. 

An enhanced chest CT showed few scattered ground-glass opacities, enlarged supraclavicular, mediastinal, and bilateral hilar lymph nodes. Differential diagnosis of TB, malignancy, and autoimmune diseases were raised. However, extensive infectious workup (including TB, bacterial, and viral), fluid cytology, and abdomen CT were negative. A broad autoimmune workup, including anti-nuclear antibodies (ANA), rheumatoid factor (RF), antineutrophil cytoplasmic antibodies (ANCA), and ribonucleoprotein (anti-RNP), was normal. The only notable finding was peripheral eosinophilia of 12%. Along with antibiotics, the patient was also started on colchicine and ibuprofen, presuming a diagnosis of idiopathic pericarditis. She was then subsequently discharged after a normal repeated echocardiogram, while continuing colchicine and ibuprofen.

Seven weeks post-discharge, she presented again with acute pleuritic chest pain, associated with palpitations and unintentional weight loss of 3 kg. She reported history of persistent dyspnoea and dry cough over the past weeks with minimal improvement with her inhalers without exposure to triggers. She denied hematuria, skin changes, neurological deficits, or family history with similar presentation.

On examination, the patient was conscious, speaking in full sentences, and afebrile. Her blood pressure was within normal limits at 96/77 millimeters of mercury (mmHg), with a mean arterial pressure (MAP) of 84. She was tachycardic, with a heart rate of 113 beats per minute, and tachypnoeic, with a respiratory rate of 30 breaths per minute. Oxygen saturation on room air was 95%. Chest auscultation revealed scattered expiratory wheezes, with normal cardiac sounds, and no pericardial rub. The jugular venous pressure was not elevated at 45 degrees, and there was no peripheral edema.

**Figure 1:**
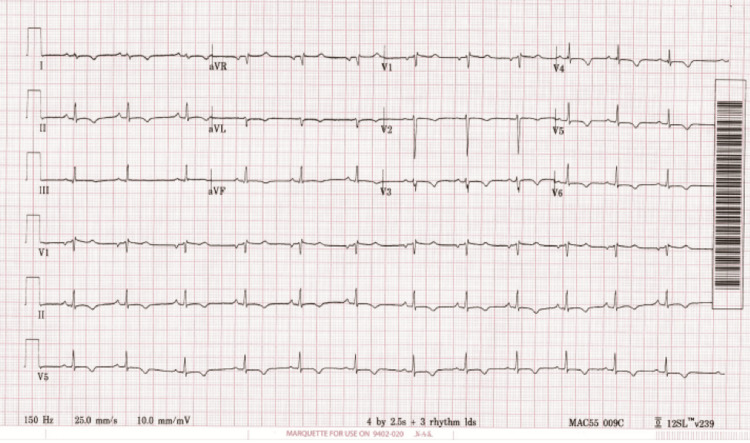
Diffuse T-wave inversion in ECG ECG: Electrocardiogram

**Figure 2:**
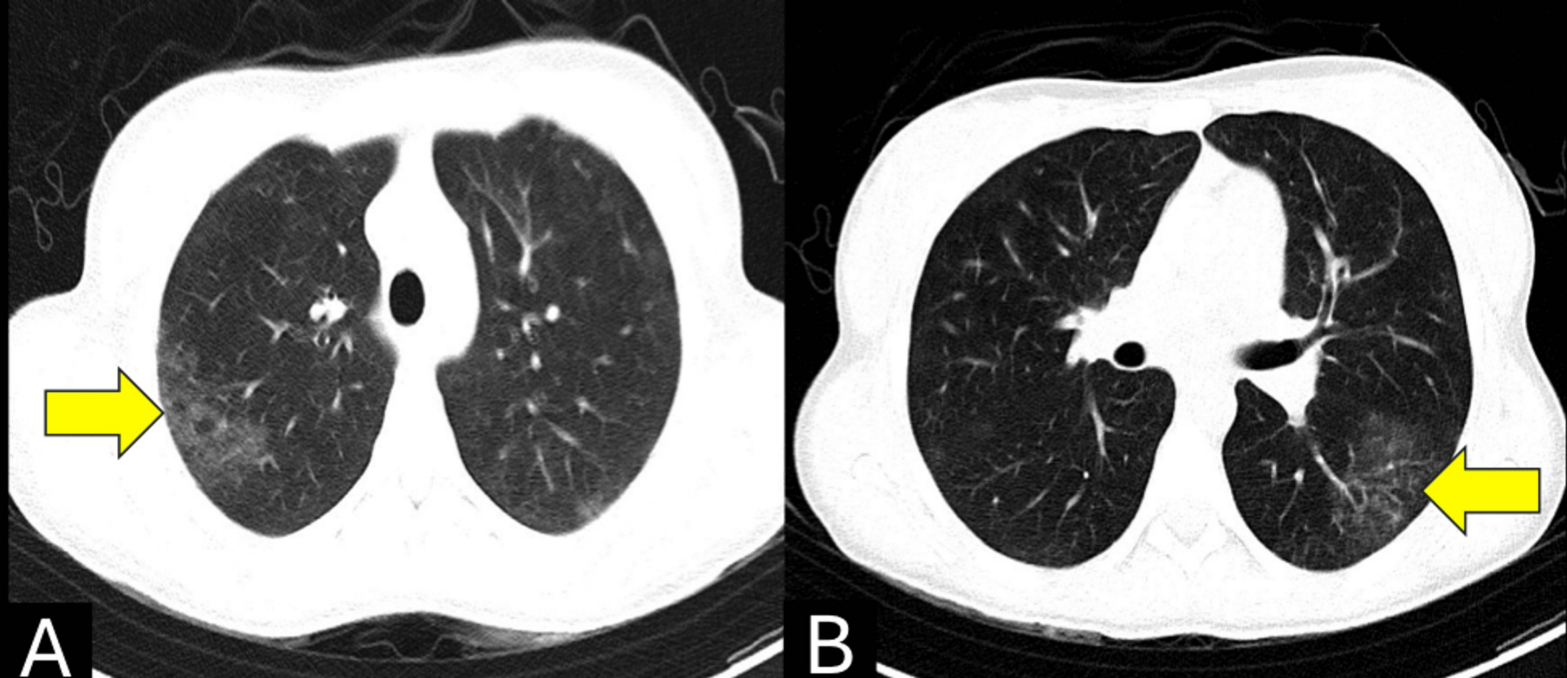
Chest CT demonstrates persistent bilateral ground-glass opacities (yellow arrows), seen seven weeks after initial presentation

Laboratory evaluation revealed leukocytosis (WBC 25.8 × 10⁹/L) and marked eosinophilia (17.9 × 10⁹/L), comprising 62% of the total count. Inflammatory markers were elevated (C-reactive protein (CRP) 31 mg/L; erythrocyte sedimentation rate (ESR) 120 mm/hr). Serum immunoglobulin E (IgE) was significantly elevated at 3,498.9 IU/mL. Repeated autoimmune serologies remained negative. Cardiac biomarkers were abnormal as troponin was 2,831 pg/mL and B-type natriuretic peptide (BNP) was 715 pg/mL. Electrocardiogram (ECG) showed diffuse T-wave inversions (Figure 1). Echocardiography demonstrated normal left ventricular function and minimal pericardial effusion. High-resolution chest CT showed persistent bilateral ground-glass opacities with no evidence of pulmonary embolism (Figure 2). Paranasal sinus CT showed no polyps.

**Figure 3:**
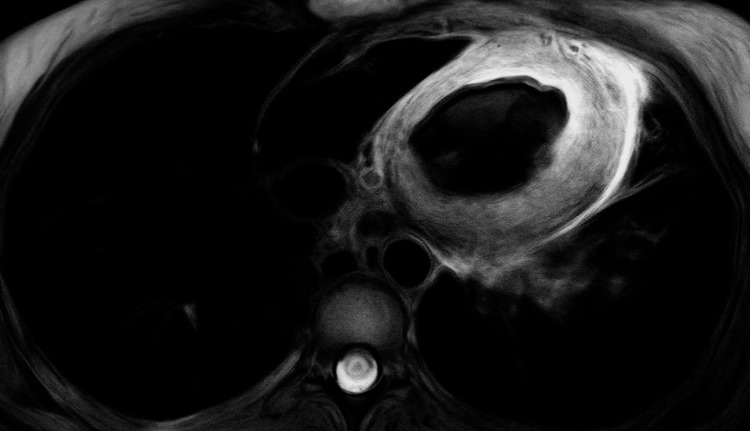
CMR with short-axis view showed extensive myocardial edema on T2-weighted fat-suppressed imaging CMR: Cardiac magnetic resonance imaging

**Figure 4:**
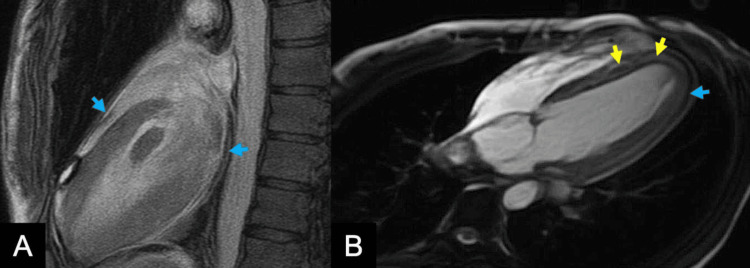
CMR with sagittal view (A) and transverse view (B) with LGE showed multifocal small areas of patchy subendocardial and mesocardial enhancement, involving basal, mid segments, and distal segments (yellow arrows). The pericardium is thickened, edematous, and enhancing with minimal pericardial effusion noted (blue arrows). CMR: Cardiac magnetic resonance imaging; LGE: Late gadolinium enhancement

Given the history of poorly controlled asthma, peripheral eosinophilia, pulmonary infiltrates, and cardiac involvement, a diagnosis of EGPA was considered. Therefore, she was started empirically on intravenous (IV) methylprednisolone. Cardiac magnetic resonance imaging (CMR) was done the following day with late gadolinium enhancement (LGE), showing results suggestive of EGPA, with normal biventricular size and preserved systolic function (Figures 3, 4). An endomyocardial biopsy (EMB) was deferred due to family preference.

Patient received a high-dose of IV methylprednisolone (250 mg daily for two days, then 500 mg for three days), followed by induction therapy with rituximab. Her symptoms and laboratory results improved subsequently, with decreased troponin to 612 pg/mL and normal eosinophil count. She was discharged on tapering oral prednisolone, three-months colchicine, and maintenance rituximab, and remained stable on follow-up.

## Discussion

EGPA, formerly known as Churg-Strauss syndrome, is an autoimmune vasculitis that involves multiple organs including the lungs, heart, skin, and peripheral nerves [1]. Recent data estimate its prevalence at 34 per million people, an increase attributed to improved recognition and awareness [6]. The most common initial presentation of EGPA is late-onset asthma, which reflects the prodromal phase, and usually precedes systemic manifestations years after diagnosis [7]. Due to the non-specific nature of asthma, diagnosis of EGPA is usually established once marked eosinophilia or systemic organ involvements are identified, which are known as eosinophilic and vasculitic phases [7]. Organ involvements are predicted mainly on ANCA-status; however, it plays a limited role in diagnosing EGPA, which essentially depends on combination of clinical features, laboratory features, and imaging studies [7]. In this case, the onset of her cardiac and pulmonary manifestations, common in ANCA-negative cases, was established only months after her asthma diagnosis, reflecting the severe progression of the disease course [1].

There are other rheumatological diseases that can present with perimyocarditis manifestations such as systemic lupus erythematosus (SLE), rheumatoid arthritis (RA), and other ANCA-associated vasculitis. Asthma, peripheral eosinophilia, and CMR findings are helpful in distinguishing EGPA [8]. Furthermore, diagnosis of EGPA is supported by classification criteria established by the European Alliance of Associations for Rheumatology (EULAR) and the American College of Rheumatology (ACR) guideline in 2022, helping differentiate EGPA from other forms of vasculitis [8]. It is a point-based system comprised of positively and negatively scored parameters. The presented case scored 8 points, which makes EGPA diagnosis with a sensitivity of 85% and a specificity of 99% [8]. These positively scored parameters include asthma, eosinophil count ≥ 1 × 10⁹/L, mononeuritis multiplex, nasal polyp, and extravascular eosinophilic-predominant inflammation. While negatively scored parameters include ANCA positivity and haematuria, but none were seen in the described case.

Cardiac manifestation, usually involving all layers of the heart, is one of the components of the Five-Factor Score (FFS) [8]. It accounts for 31% of EGPA deaths; hence, early recognition is critical [9]. A meta-analysis, highlighting the initial cardiac features that led to EGPA diagnosis in 62 cases, found around 17% had subclinical features, characterised only by abnormal laboratory and imaging findings [3]. 32% and 37% of the cases presented with chest pain and mild pericardial effusion, respectively [3]. Although EGPA pericardial effusion is often reported as exudative with marked eosinophils, it can occur as mild hemorrhagic pericardial effusion as reported by Arinaga et al., after excluding other causes such as malignancy, infection, trauma, and other immunologic diseases, all of which were excluded in the presented case [1,10,11]. Pericardial effusion in EGPA are usually reported as mild [5]. However, a rare case of isolated cardiac tamponade was reported by Alam et al., where a patient was diagnosed with EGPA solely based on large exudative eosniphilic pericardial effusion, marked peripheral eosinophilia, with no other organs involved, a diagnosis that was then confirmed by pericardial biopsy [12]. In the described case, diagnosis of EGPA was delayed due to underrecognition of her initial large bloody pericardial effusion, which prompts us to highlight this rare presentation. 

Perimyocarditis, characterized by extension of pericarditis to the myocardium, accounts for 13.6% of cardiac manifestations in EGPA patients [2]. It is diagnosed by increased troponin level, diffuse T-wave inversion, and some distinct CMR findings [13]. Once EGPA-induced myocardial injury is suspected, it is important to perform multiphasic imaging such as CMR, especially if EMB is unavailable as in the presented case [14]. CMR in this case demonstrated multifocal, nonterritorial areas of patchy subendocardial and mesocardial LGE. T2-weighted imaging showed extensive myocardial edema, and the pericardium appeared thickened and edematous. These features, combined with elevated troponin and diffuse T-wave inversion on ECG, are cardinal findings consistent with perimyocarditis in EGPA [7,13,15]. CMR can also be detected early in those with subclinical features of EGPA, highlighting its high sensitivity in comparison to other modalities [2,7]. CMR is also used to detect treatment efficacy and extension of fibrosis in patients with EGPA [7].

Early treatment is crucial to control disease activity, minimize organ damage, and improve prognosis. The multisystem nature of vasculitis necessitates a collaborative management approach across specialties of rheumatology, neurology, and cardiology to ensure optimal outcomes. Current guidelines recommend to treat EGPA based on severity classification [8]. For organ-threatening cases such as cardiac ones, it is recommended to start pulsed IV glucocorticoids (500-1000 mg of methylprednisolone daily for three days), followed by high-dose oral glucocorticoids (0.75-1 mg/kg per day). Cyclophosphamide (every two weeks for one month, then every four weeks at 0.6 g/m² per dose) or rituximab (1 g pulses two weeks apart) are used in combination to induce remission in severe cases [8]. To sustain disease control and prevent relapse, adjunctive immunosuppressants are recommended for maintenance therapy in severe cases such as rituximab, mepolizumab, or azathioprine with tapering doses of oral glucocorticoids [8]. In the presented case, rituximab was selected for induction over cyclophosphamide due to the patient's young age and the need to avoid possible gonadotoxic effect and risk of infertility associated with cyclophosphamide. The patient responded well with rituximab and IV steroid, with maintenance rituximab, and no relapse has been detected to date. 

## Conclusions

We presented a rare case of EGPA perimyocarditis evolving after hemorrhagic pericardial effusion. Cardiac manifestations are common in EGPA, yet early detection is still challenging, and it carries significant prognostic value. Diagnosis of EGPA is based on a combination of clinical, laboratory, and imaging findings, supported by EULAR/ACR 2022 criteria. Large hemorrhagic pericardial effusion is rarely reported in the literature in EGPA, and other causes have to be excluded. CMR is a cardinal non-invasive tool in assessing involvement of perimyocarditis in EGPA. Treatment with cardiac involvement requires pulse IV steroid with immunosuppressants such as rituximab with maintenance of immunosuppressive medications. This case highlights the importance of considering EGPA in the differential diagnosis of hemorrhagic pericarditis, especially in young patients with unexplained eosinophilia, and emphasizes the importance of early recognition of these cardiac manifestations for better outcomes. In addition to continued monitoring and long-term follow-up to detect disease relapses and guide adjustments in immunosuppressive therapy. 
